# *Marvellous to mediocre*: findings of national survey of UK practice and provision of care in pregnancies after stillbirth or neonatal death

**DOI:** 10.1186/s12884-016-0891-2

**Published:** 2016-05-06

**Authors:** T. A. Mills, C. Ricklesford, A. E. P. Heazell, A. Cooke, T. Lavender

**Affiliations:** School of Nursing, Midwifery and Social Work, The University of Manchester, Room 4.334, Jean McFarlane Building, Oxford Rd, Manchester, M13 9PL UK; Institute of Human Development, The University of Manchester, Manchester, UK; Central Manchester Foundation NHS Trust, Manchester, UK; Manchester Academic Health Sciences Centre, Manchester, UK

**Keywords:** Stillbirth, Neonatal death, Subsequent pregnancy, Antenatal care, Maternity services, Womens’ experiences

## Abstract

**Background:**

Pregnancy after stillbirth or neonatal death is an emotionally challenging life-event for women and adequate emotional support during pregnancy should be considered an essential component of quality maternity care. There is a lack of evidence surrounding the role of UK maternity services in meeting womens’ emotional and psychological needs in subsequent pregnancies. This study aimed to gain an overview of current UK practice and womens’ experiences of care in pregnancy after the death of a baby.

**Methods:**

Online cross-sectional surveys, including open and closed questions, were completed on behalf of 138 United Kingdom (UK) Maternity Units and by 547 women who had experience of UK maternity care in pregnancy after the death of a baby. Quantitative data were analysed descriptively using SPSS software. Open textual responses were managed manually and analysed using the framework method.

**Results:**

Variable provision of care and support in subsequent pregnancies was identified from maternity unit responses. A minority had specific written guidance to support care delivery, with a focus on antenatal surveillance and monitoring for complications through increased consultant involvement and technological surveillance (ultrasound/cardiotocography). Availability of specialist services and professionals with specific skills to provide emotional and psychological support was patchy. There was a lack of evaluation/dissemination of developments and innovative practice. Responses across all UK regions demonstrated that women engaged early with maternity care and placed high value on professionals as a source of emotional support. Many women were positive about their care, but a significant minority reported negative experiences. Four common themes summarised womens’ perceptions of the most important influences on quality and areas for development: *sensitive communication and conduct of staff*, *appropriate organisation and delivery of services*, *increased monitoring and surveillance* and perception of *standard vs. special care*.

**Conclusions:**

These findings expose likely inequity in provision of care for UK parents in pregnancy after stillbirth or neonatal death. Many parents do not receive adequate emotional and psychological support increasing the risk of poor health outcomes. There is an urgent need to improve the evidence base and develop specific interventions to enhance appropriate and sensitive care pathways for parents.

## Background

In the UK around 15 babies a day die shortly before or soon after birth [1]. The extent and impact of stillbirth or neonatal death on parents remains widely misunderstood and consequently grief is often disenfranchised [2]. The majority of women conceive again, 50 % within a year of the loss [3] and subsequent pregnancies are consistently associated with increased maternal anxiety and emotional vulnerability [4]. Psychological distress in pregnancy is acknowledged as a major public health issue and associated with a range of adverse outcomes including preterm birth and low birth weight [5]. Furthermore, persistence of negative psychological impacts even beyond the subsequent birth is related to disrupted maternal attachment and parenting difficulties [6]. This evidence suggests that appropriate psychological support ought to be considered a key component of quality care in pregnancy after stillbirth or neonatal death.

The Royal College of Obstetricians and Gynaecologists (RCOG) recommends obstetric-led care and birth for the majority of women pregnant after perinatal loss due to the increased risk of recurrence [7]. Current guidance contains little practical advice on emotional and psychological support for parents [8]. In the absence of a defined pathway, care is likely to vary according to the interest and expertise of individual clinicians. Our recent metasynthesis of the literature raised important issues surrounding experiences of care in pregnancy after perinatal death [9]. Emotional turbulence isolated parents from usual support networks of friends and family and tended to increase dependence on professionals for emotional support in pregnancy. However, parents perceived an over-emphasis on technology e.g. ultrasound scans and fetal monitoring to provide reassurance and a lack of focus on interpersonal relationships. Most of the included studies were conducted focussed on North America, where maternity care is obstetrician-dominated and midwives have a lesser role, potentially limiting applicability to the UK context. As part of a programme of exploratory work, focused on in-depth understanding of parents’ needs, this study reports an overview of UK service provision and the views of women on their pregnancy care after stillbirth or neonatal death.

## Methods

Cross-sectional online surveys were conducted to explore current UK provision and practice of care in pregnancy after stillbirth or neonatal death. A favourable ethical opinion was received from National Research Ethics Service (NRES) Committee North West – Greater Manchester North (Ref 11/NW/0806) and the University of Manchester Ethics Committee.

### Sample

To gain an overview of current service provision and care pathways for women in pregnancy after stillbirth or neonatal death, all UK maternity units were approached to complete a questionnaire. Women who were currently pregnant, or had previous experience of maternity care following the stillbirth or neonatal death of their previous baby were invited to respond to a separate survey exploring their experiences. Electronic links were sent via email to the Heads of Midwifery at 188 units, where necessary contact details were verified with relevant Nursing and Midwifery Council (NMC) Local Supervising Authority. Where no response was obtained, reminders were sent monthly until the close of the survey. Advertisements inviting women to participate were placed on the *Sands* (Stillbirth and neonatal death charity), *Tommy’s* (Pregnancy research charity), *BLISS* (Premature and sick baby charity) *Netmums, babycentre.com and Bounty* (Parenting advice and information) websites, with a link, information about the study and contact details for the research team for any questions related to participation. Consent was confirmed by the participant completing and submitting or returning the questionnaire. Four women requested a paper version of the survey and returned these via post. Surveys remained open for a six month period, ending late in 2012 to optimise maximum variation of the sample.

### Questionnaires

The questionnaires were designed following a systematic review and metasynthesis of the qualitative literature which identified key themes arising from parents’ experiences of care in pregnancy after stillbirth or neonatal death [9] and further informed by a survey of bereaved parents conducted by a member of the research team [10]. The topic areas selected were confirmed in discussions with the study Stakeholder Group, which included clinicians, recent service users and support group members (Manchester Sands) and multidisciplinary clinicians thus gaining content validity. The questionnaire for maternity units included mainly fixed-response questions, focused on four areas; unit guidelines and policies, information and support for women, bereavement specialist midwife involvement and service development with free text boxes to explain any ‘other’ responses and describe new care provision. The women’s questionnaire included fixed-response and Likert- style questions [11, 12] related to the death of the previous baby, the timing of the next pregnancy, antenatal booking and the first appointment, subsequent appointments, contact with services outside scheduled appointments and information and support within and outside the NHS. In addition, three free-text questions (Table 1) allowed women to further elucidate their views and experiences and include other information they considered important. Face validity, comprehensibility and ease of online completion were assessed by a panel of professionals experienced in caring for women after stillbirth and neonatal death and subsequent pregnancies, including eight midwives and two obstetricians. Where appropriate, questions were amended to improve clarity.

**Table 1:** Open text question used in the women’s survey

Q24	Do any of your interactions with health professionals, listed above, or others stand out? If yes, please describe what happened and what was important for you?
Q25	Was there anything particularly good or bad about the care you received in the pregnancy following the death of your baby? If so, please describe
Q 31	Are there any changes you feel would improve the service you received?

### Analysis

Responses were de-identified, assigned a participant number and electronically password protected to ensure confidentiality. Quantitative data were entered into SPSS 20 (SPSS Inc. Chicago, IL, US) and analysed descriptively. The free-text responses to the three questions, shown in Table 1, were analysed by two researchers (CR/TM), independently, and initially categorised as either describing experiences which were *positive*, *negative* or *mixed* (including both positive and negative facets). In two cases there was disagreement over the initial assessment (one negative/mixed (finally categorised as mixed) and one mixed/positive (finally categorised as positive) and, consensus was reached after re-reading and further discussion. The textual content was analysed using the framework method [13]. Five phases of familiarisation, developing a theoretical framework, charting, summarising, mapping and interpretation as described in detail by Ward et al [14] were used to uncover prominent themes in the data. Interpretation was then confirmed in discussion with the wider research team. In addition, a summary of the main themes was presented to the study Stakeholder Group for additional feedback and discussion.

## Results

### Response rates

The maternity unit survey attracted 184 responses, after exclusion of duplicates (multiple responses from same unit; 43) and responses which were less than 50 % complete (3) the effective response rate was 73 % (138 units). For women, 802 responses were received. Responses were removed if the woman had not had a pregnancy after the stillbirth or neonatal death (129), if all pregnancy losses occurred prior to 22 weeks gestation (World Health Organisation [WHO] definition of stillbirth; 46) or less than 50 % of the survey was completed (78), leaving 547 for further analysis.

### Demographics

The characteristics of participating maternity units and women are shown in Tables 2 and 3. Responses were received from women across all geographical regions of the UK and a range of maternity settings, from large tertiary obstetric units to stand-alone birth centres. For the women’s survey, the majority of respondents were over 30 years (71.3 %) and White although a small number of Indian, Afro-Caribbean, Bangladeshi, Pakistani, African and Far Eastern women (total Black and minority ethnic [BME] women 14 [2.7 %]) participated. Although no specific time limit for the loss or subsequent pregnancy was imposed for participation, 87 % of women had experienced their most recent stillbirth or neonatal death (2 % had more than one stillbirth or neonatal death) within the previous ten years, with 60.9 % of the baby’s deaths occurring in the last five years. Therefore, the majority of experiences reported in this study will relate to contemporary maternity care. Similar to previous reports [3], the inter-pregnancy interval was relatively short (Table 3), 86 % of women reported becoming pregnant within 18 months of the death of their baby and the overwhelming majority of pregnancies were planned.

**Table 2:** Demographic characteristics of maternity units (*N* = 138)

Characteristic		N (%)
Geographical location		
	Scotland	18 (13 %)
	North East England	7 (5.1 %)
	North West England	22 (15.9 %)
	Yorkshire and Humber	6 (4.3 %)
	East Midlands	8 (5.8 %)
	West Midlands	13 (9.4 %)
	East of England	12 (8.7 %)
	London	11 (8 %)
	South East England	15 (10.9 %)
	South West England	16 (11.6 %)
	Northern Ireland	7 (5.1 %)
	Wales	3 (2.2 %)
Type of unit		
	Tertiary centre	29 (21 %)
	District general hospital	103 (74.6 %)
	Birth centre/Midwife-led unit	6 (4.3 %)
Births per year		
	>5000	42 (30.4 %)
	3000–5000	54 (39.1 %)
	1000–3000	34 (24.6 %)
	<1000	8 (5.8 %)

**Table 3:** Demographic characteristics of women participants (*N* = 547)

Characteristic		N (%) or median (range)
Age range		
	Up to 20	6 (1.1 %)
	21–30	151 (27.6 %)
	31–39	302 (55.2 %)
	Over 40	88 (16.1 %)
Ethnic group	White British/Irish/Other	526 (96.2 %)
Geographical location		
	Scotland	69 (12.6 %)
	North East England	20 (3.7 %)
	North West England	60 (11.0 %)
	Yorkshire and Humber	41 (7.5 %)
	East Midlands	39 (7.1 %)
	West Midlands	49 (9.0 %)
	East of England	30 (5.5 %)
	London	42 (7.7 %)
	South East England	103 (18.8 %)
	South West England	58 (10.6 %)
	Northern Ireland	9 (1.6 %)
	Wales	27 (4.9 %)
Year of most recent stillbirth/neonatal death		
	< 2003	70 (12.8 %)
	2003–2007	144 (26.3 %)
	2008–2012	333 (60.9 %)
Type of death		
	Stillbirth	400 (73.1 %)
	Neonatal death	147 (26.9 %)
	First baby	331 (60.5 %)
Gestation (weeks)		34 (22–42)
Inter-pregnancy interval (months)		6 months (1 month-16 years)
Subsequent pregnancy planned		479 (88 %)

### Maternity unit survey

Concerning their current provision for pregnant women who had experienced a previous stillbirth or neonatal death (Table 4), 40 % of units reported having some written guidelines to support care. Only one unit supplied a copy to the research team, this included points within a guideline related to care after stillbirth rather than a separate document. Where guidelines existed, monitoring and surveillance appeared to be accorded more emphasis than emotional support and psychological care. For example, few included specialist midwife input, access to counselling or specialist antenatal education to prepare for the birth and transition to parenthood.

**Table 4:** Availability of policies, guidelines or pathways for care in subsequent pregnancies

		N units (%)
Unit has a written policy, guideline or pathway:		
	Yes	55 (39.9 %)
	No	80 (58.0 %)
	Don’t know	3 (2.2 %)
Policy/guideline/pathways includes:		
	Consultant involvement	44 (80 %)
	Early Scan	31 (56.4 %)
	Additional antenatal appointments	31 (56.4 %)
	Additional ultrasound	27 (49.1 %)
	Specialist midwife (e.g. bereavement midwife) involvement	20 (36.4 %)
	Bereavement counselling	9 (16.4 %)
	Specialist antenatal education	6 (10.9 %)
	Other	3 (5.5 %)

Anecdotal reports suggested that specialist bereavement midwives often provided care and support to previously bereaved families, therefore the availability of a bereavement midwife and her role in caring for women subsequent pregnancies was probed (Table 5). Half of the responding units had a bereavement midwife in post but their involvement varied. In a minority of units, bereavement midwives were formally assigned to provide antenatal care in subsequent pregnancies, but more frequently their input was *ad hoc.* However, only 13 % of units reported that their bereavement specialist midwife had no involvement with women in subsequent pregnancies. Specific service developments were reported by 22 units (18 %) including offering continuity of midwifery care and caseload models, involvement of the bereavement midwife and referral to specific consultants, support groups, some with third sector organisation involvement e.g. Sands. One unit had a dedicated antenatal clinic. Formal evaluation of new innovations was reported by 5 units, but none of the evaluations were supplied to the research team or were publically available for dissemination.

**Table 5:** Availability of specialist support in pregnancy after stillbirth or neonatal death

		N units (%)
Bereavement specialist midwife in post :		
	Yes	60 (49.6 %)
Role in antenatal care		
	Formal allocation	17 (29.7 %)
	Ad hoc involvement	
	*Telephone support only*	18 (29.5 %)
	*Antenatal care/Telephone support*	15 (24.6 %)
	*Antenatal care*	5 (3.6 %)
	*No involvement*	8 (13.1 %)

### Women’s survey

The majority of women (440; 80.4 %) returned to the same maternity unit they attended when their previous baby had died; the median gestation at first appointment in the subsequent pregnancy was 8 weeks (range 3-24 weeks). For those who went to different hospitals (107; 19.6 %), the primary reason stated was a perception of poor care surrounding their previous baby’s death (43; 40.2 %) or painful memories associated with the setting (23; 21.5 %). However, women also changed hospitals because they moved into a different area (21; 19.6 %) or to access specialist care as a result of identified maternal or fetal clinical indications (14; 13.1 %). Women saw a range of professionals at their first appointment, with 42.2 % (231) recalling seeing a consultant obstetrician. However, only 51.7 % (283) women felt at least *‘well prepared’* for their pregnancy after attending their first antenatal visit.

Regarding subsequent appointments, 73.5 % of women (405) considered that the frequency was appropriate, although 21.9 % (120) wanted to be seen more often, only two women believed that they had too many antenatal appointments. Reflecting the maternity unit survey, the majority of women (430; 78.6 %) confirmed that a consultant obstetrician had been involved in their antenatal care, many also had additional surveillance including extra ultrasound scans (409; 74.7 %), additional appointments (375; 68.5 %) and were offered an early pregnancy *‘viability’* ultrasound (327; 59.7 %). Fewer women reported input from specialist midwives (69; 12.6 %), attending standard or specialist antenatal classes (58; 10.6 %) or accessing bereavement counselling (45; 8.2 %).

History of perinatal loss has been associated as a factor increasing health care utilisation amongst pregnant women [15]. Therefore, women were asked to recall additional advice or contacts outside scheduled antenatal appointments (Table 6). Many women contacted health professional, frequently via hospital maternity triage or day units, with concerns about their own or the baby’s health during pregnancy often on multiple occasions. Non-healthcare support and information was accessed by 41.9 % of women during pregnancy after stillbirth or neonatal death (Table 7) and generally highly valued, 95.6 % (219) of women rated the sources they used as helpful or very helpful.

**Table 6:** Contacts with health services outside scheduled appointments

			N women (%)
Contact with health services between regular appointments:			
	Yes		311 (56.9 %)
		No of contacts^a^	
		1	52 (16.7 %)
		2	75 (24.1 %)
		>2	182 (58.5 %)
	No		214 (39.1 %)
	No answer		22 (4 %)
Service contacted:^b^			
	Maternity triage/assessment unit		151 (48.5 %)
	Maternity day unit		119 (38.2 %)
	GP		105 (33.7 %)
	NHS direct		20 (6.43 %)
	Other^c^		79 (25.4 %)

^a^% of women who responded ‘yes’

^b^% totals more than 100 as respondents could select more than one response

^c^Included direct contact with community midwives or consultant obstetricians (28; 9 %) and private sector providers (11; 3.5 %), particularly for additional ultrasound scans

**Table 7:** Support outside healthcare in pregnancy after stillbirth or neonatal death

		N women (%)
Accessed non-healthcare support or information :		
	Yes	229 (41.9 %)
Sources		
	Sands Groups/Befrienders^a^	119 (21.8 %)
	Online Chat forums	96 (17.6 %)
	Websites	95 (17.4 %)
	Telephone helplines	24 (4.4 %)

^a^Trained peer supporter

### Womens’ experiences and perceptions of care in subsequent pregnancies

Of the total 547 respondents, 336 (61.4 %) and 411 (75.1 %), respectively, answered free text questions 24 (Q24) and 25 (Q 25; Table 1). Of these, a small number (25 for Q 24; and 18 for Q 25) were a single word ‘No’. ‘No’ was interpreted as meaning that no interaction stood out and nothing was particularly good or bad about the care received, respectively. For question 31 (Table 1), of 489 (89.4 % of total) responses, 327(76.3 %) women felt that some changes to services were needed to improve care for parents in pregnancy stillbirth or neonatal death.

A majority of the responses described were positive experiences of care, especially in answer to question 24, although a substantial number of negative encounters were also reported, more frequently to question 25 (Table 8). Positive experiences most frequently involved consultant obstetricians, followed by midwives and bereavement specialist midwives. Trainee doctors tended to feature more prominently in negative encounters although midwives, consultants and sonographers were also cited. There was substantial overlap in womens’ responses and four common themes were identified summarising the most important influences on care and areas identified for development namely; *sensitive communication and conduct of staff*, *appropriate organisation and delivery of services*, *increased monitoring and surveillance* and perception of *standard vs. special care*.

**Table 8:** Categorization of free text responses

Category	Q .24 (*N* = 336)	Q.25 (*N* = 393)
Positive	252 (75 %)	187 (46 %)
Negative	38 (11 %)	153 (37 %)
Mixed	21 (6 %)	53 (13 %)

### Sensitive communication and conduct

Explicit recognition by health professionals of the need for increased emotional support in pregnancy after stillbirth or neonatal death, in addition to monitoring and physical care, was a crucial element in positive perceptions for participants:

*‘ Consultant - she seems to care about the risks involved and stress we are under…my previous birth experience was exactly the opposite…..’ P 37*

It was important that professionals demonstrated empathy and understanding of the impact of past experiences on the current pregnancy. Open acknowledgment of the death of the previous baby, anticipation, understanding and a compassionate response to anxiety and fluctuating emotions were all highly valued:

*[Consultant]… ‘Talked about my first baby, remembered her name.’* P 576

Negative experiences were frequently characterised by communication failures. Numerous accounts recalled professionals, including midwives and obstetricians, being unaware of the woman’s history and apparent lack of reading notes. Many women described having to prepare themselves for uncomfortable questions at appointments:

*‘The main upset was having to explain every time to different midwives my situation because it was someone different each time and they hadn't read the notes so would ask the same questions, “I bet you’re excited” answer “no, not excited just petrified of losing my baby…….’* P 311

Devices designed to alert professionals to previous stillbirth or neonatal death, such as prominent special stickers (applied to front of case/handheld notes with women’s consent) were not always recognised or acknowledged in practice:

*‘At my first NHS scan I became very distressed, the sonographer asked why I was so upset and I asked if she had read my notes, she hadn't so we had to explain our history despite having provided my own Sands stickers and ensured they were on my handheld notes and hospital notes. I was upset that despite trying to ensure it would be clear that we had suffered the loss of our baby that at our very first appointment we had to explain ourselves.’ P 165*

Knowledge of the woman’s previous experience did not always guarantee sensitive behaviour and communication. Some professionals exposed a lack of knowledge and understanding of the continuing impact of perinatal bereavement and poor interpersonal skills during contacts with bereaved parents:

*A bad experience was when a midwife on MDU* [maternity day unit] *commented that women who receive extra care following a stillbirth such as the planned CTG* [cardiotocograph] *I had did not medically need to be seen. The implication was that we were wasting her time which upset me*. P 285

Lack of effective listening skills and a tendency to make assumptions were major barriers to effective communication and the development of relationships;

*Midwife didn't know about my set of circumstances or have the time or inclination to find out. She always assumed my subsequent pregnancy was worrying and stressful, which was frustrating as on the whole I was enjoying it! She just didn't seem able to actually listen to what I was saying-in that typical health care practitioner’s way!* P 124

Improving communication, specifically ensuring that all staff were aware of parents’ history before contact was the most frequent recommendation for improving relationships with professionals. Women also acknowledged the importance of education and training for staff to better understand the impact of bereavement and the necessity of individualised emotional support alongside physical monitoring.

### Appropriate organisation and delivery of services

Aspects of organisation and delivery of services also featured prominently in womens’ responses. Having continuity of carer (particularly from midwives and obstetricians) was consistently linked with positive experiences and perceptions. Several women opted to be cared for by the same professionals as during the previous pregnancy which ended in the death of their baby:

*As well as my consultant appointments I saw the same community midwife as in my previous pregnancy. As my pregnancy progressed and my anxiety levels heightened she saw me every week to offer reassurance.* P 39

Contact with a small number of professionals from early pregnancy enabled women to develop supportive and trusting relationships. They also felt empowered to take an active role in planning their care including timing and mode of birth.

Flexibility in scheduling, providing extra appointments on request and ensuring women had access to effective advice and support between appointments were also highly valued;

*…. community midwife very attentive throughout and happy to see us anytime. Came to see us at home on more than one occasion to talk things through. Also, twice weekly CTG* [cardiotocographs] *at ADU* [Antenatal Day Unit] *for reassurance.* P 496

Women expressed considerable dissatisfaction with traditional models of hospital antenatal care, where they encountered different midwives and frequently trainee doctors, at each visit. The absence of meaningful relationships with care providers impaired communication and limited opportunities to offer emotional support. For several women these visits increased rather than alleviated anxiety and vulnerability.

*I found the hospital antenatal appointments extremely hard because I had to see about 4 different professionals each visit (other clerk, assistant to take BP, phlebotomist? to take blood, registrar and sometimes sonographer). Each time I had to run the gamut of meeting these people and them potentially saying something insensitive because they didn't know my background. It also meant that I got less psychological support than if these checks had been done by a community midwife who I had a relationship with. It was production line medicine rather than person centred care*. P 299

Assessment of individual needs and preferences was of paramount importance in planning sensitive care. A few women were unhappy at being allocated to the same professionals who were involved in their previous pregnancy without prior discussion:

*..I hated having the same consultant and it never crossed my mind to ask for someone else. I’m sure it was felt consistency would be a good thing but I was unhappy with the care I received from that consultant when my son died.* P 124

Women felt strongly that continuity, being cared for by known and empathetic midwives and senior obstetricians at all their antenatal appointments, was important. Being consulted over which professional they would see was important in some circumstances. Many women also wanted the opportunity to have access to specialist providers who were perceived as having unique skills and experience to provide emotional support; bereavement specialist midwives and counsellors were most frequently mentioned. Input into planning care and discussion of the frequency of appointments and ultrasound scans was important. Environmental considerations particularly the stress and anxiety raised by long waiting times and having to use the ‘general’ waiting areas in antenatal clinics were also raised.

### Careful monitoring and surveillance

Unsurprisingly, women were very focussed on ensuring that they received thorough monitoring for complications and regular reassurance of fetal well-being throughout pregnancy. Increased frequency of visits and technological surveillance through ultrasound scans and cardiotocograph monitoring (CTG) were expected and generally valued as a source of reassurance:

*Midwife has been excellent, listened to my worries and has been very cautious about certain things, even sending me to hospital for extra scans and monitored blood pressure checks. Have felt very reassured under her care.* P166

Regular personal input from a senior experienced professional, usually the consultant obstetrician, was also regarded as important by many women.

Care did not always meet women’s expectations; some women felt that they should have been offered more frequent ultrasound scans and monitoring in late pregnancy. Difficulty in accessing an early viability ultrasound scan, which can be performed at 6–10 weeks gestation to establish normal progress of pregnancy and the number of fetuses, was also a recurrent issue:

*I have been pregnant 3 times since my son died. 2 miscarriages at 11 weeks and I am now 7 week*[s]*. So far what stands out is lack of support. Nobody will see me or refer me for a reassurance scan. Each pregnancy has been a struggle to get appointments. I have been told by my consultant I need to be seen before 12 weeks but just cannot get the appointments.* P 479

### ‘Special’ vs ‘Standard’ care

Given the poor previous outcome, it was important to many women that they were able to identify perceptible differences in the package of care offered during their subsequent pregnancy. Views on ‘appropriate’ or ‘ideal’ care varied between participants, but for most it extended beyond a few extra appointments or additional ultrasound scans. Women valued building a relationship with empathetic and compassionate professionals during their pregnancy. Trust and mutual respect provided reassurance that everything possible was being done to improve the outcome for this pregnancy. This, described by one woman as, ‘Rolls Royce’ care had a profound and lasting impact on women and families:

*Our Midwife in our subsequent pregnancy was the same midwife who delivered our stillborn daughter. She was simply amazing. Nothing was too much trouble. It was like having a personal midwife. She even came out to me one evening (around 10 pm) when I was concerned at lack of movement to reassure me. We will never forget her kindness and dedication.* P41

In contrast, the perception of lack of any extra care was a common source of dissatisfaction. Women believed that in being *‘treated as any other mum.’* (P205) the impact of the death of their baby was not recognised. Several women linked this to not having an identified reason why the baby had died or any other medical complications in their pregnancy. Women described a ‘fight’ to get additional care they perceived as necessary, conflict with health professionals compounded anxiety.

Parent education and preparation for the birth were cited as lacking appropriate provision, women recounted poor experiences in both hospital and private-provider classes. They felt that content was often not appropriate and worried about asking questions or sharing experiences in groups with other parents who had little knowledge or understanding of poor outcomes:

*When I did the tour of the hospital as it was a new hospital I went on regular tour this was extremely hard as I had to watch whilst they explained water births etc which was not relevant to me, also they explained what happened in an emergency and the resuscitation trolley this was extremely hard to listen to, I think I should have had a separate tour. I didn’t want to ask questions in case I frightened the first time mums.* P 279

Specialist provision was highlighted as a potential area for service development; several women requested more targeted antenatal education and emphasis on preparation for the birth. Some also would have valued the opportunity to meet other parents with similar experiences through a pregnancy peer support group.

## Discussion

These results represent the largest study describing current provision and womens’ views of care in pregnancy after stillbirth or neonatal death in the UK. Units confirmed that limited formal guidance was available to support clinical staff in providing care to women in pregnancy after stillbirth or neonatal death, reflecting the lack of evidence-based care pathways in this area. Where guidelines existed, the focus was prevention of recurrence through detection of complications rather than psychological well-being and emotional support for parents. The availability and involvement of professionals with specialist skills and knowledge in bereavement care described was patchy. Development of services and dissemination of innovative practice was restricted. Despite these barriers, many women were extremely positive about the care they had received, testified by examples of empathetic and compassionate practice. However, there was also evidence of inequity; not all women received adequate support and some described very poor experiences which undoubtedly compounded anxiety and increased the risk of poor psychological outcomes.

The results of this study highlight striking parallels in bereaved women’s experiences of care across countries and healthcare systems. Qualitative data from North America [16, 17] and recent UK studies [18] also identified the emphasis placed on professionals as a key source of emotional support and reassurance by women who were pregnant after stillbirth or neonatal death. This is likely to stem from the high levels of worry, fear and uncertainty women experienced and isolation resulting from a general lack of social validation of the sequelae of perinatal bereavement. However, antenatal contacts often failed to meet expectations. Consistent with previous studies [19], communication failures, stemming from lack of awareness of women’s history or understanding of the impact of the baby’s death were major factors in dissatisfaction with care. Negative encounters involved professionals across a range of backgrounds (obstetricians, midwives and allied professions) however, junior staff (particularly obstetricians) were mentioned more frequently raising questions around pre-registration education and support for professionals at an early stage of their careers.

The survey results suggest that psychosocial care for parents in subsequent pregnancies after stillbirth or neonatal death might also be limited in the absence of professionals with specialist training and experience. In common with previous reports [20] bereavement specialist midwives were not ubiquitous across UK services and their roles and responsibilities in supporting women in their subsequent pregnancy were variable. Nevertheless, women who had input from a bereavement midwife in their subsequent pregnancy were almost universally positive about the impact of this care on their experience. Anecdotal evidence also supports a role for bereavement specialist midwives in training and supporting staff. While the beneficial effect of bereavement midwife care for parents in subsequent pregnancy has not been conclusively established, improved outcomes for women who received midwife-led support counselling and debriefing interventions after traumatic birth have been confirmed in randomised trials [21, 22]. Further development of this role, including nationally agreed standards for training and specification of responsibilities will need to be considered to ensure equity in delivery of services [23].

Although the conduct and behaviour of individual professionals was undoubtedly a significant determinant of the quality of care in subsequent pregnancy, this study also highlighted issues surrounding service organisation and delivery. Many women described rarely seeing the same obstetrician and midwives at their antenatal appointments. This lack of continuity was perceived as depriving women of the opportunity to develop relationships with professionals and as a direct cause of distressing communication breakdowns. Such fragmentation is acknowledged as negatively impacting on the overall quality of maternity care and is consistently associated with decreased satisfaction and increased intervention [24]. The relationship with the midwife, who has the most direct and intimate contact with the mother, may be particularly important in promoting adequate emotional support for women in pregnancy [25]. Randomised controlled trials of continuity of midwifery care including ‘caseholding’ models have demonstrated improved clinical outcomes and cost effectiveness for low and mixed risk women [26, 27], although women with a history of perinatal death were excluded from participation in many of these studies.

Strengths of this study include the high response rate of 73 % of maternity units, considered within the optimum range for clinical survey research [28] and the broad geographical spread of participants from all UK regions. Consistent with trends in health surveys, the generalisability of results is reduced by the under-representation of women from black and minority ethnic groups [29]. Recruitment of participants via charity and support groups could also be viewed as limiting generalisability, younger and BME parents and those with lower socio–economic status or educational attainment are less likely to access third sector websites or join support groups than affluent white women [30]. The experiences and outcomes for these parents, and other socially disadvantaged groups (e.g. refugees and asylum seekers, poor mental health and learning disability) in pregnancy after stillbirth or neonatal death are also likely to be further negatively impacted by inequalities in access to existing health services [31]. Therefore important evidence surrounding the specific needs of these often vulnerable women remains lacking. This acknowledged limitation and recent data highlighting that Black or Black British, Asian or British Asian women have 50 % increased risk of perinatal death compared with the general population [1], emphasise the urgent need to specifically explore the experiences of socially disadvantaged women and other ‘hard to reach’ groups in pregnancy after stillbirth or neonatal death [32]. The perspectives of fathers were also not directly addressed in this study.

## Conclusion

Improving quality of maternity care and outcomes for mothers and babies is a key element of UK Government Policy [33]. Although this study demonstrates many examples of good practice, the experiences of significant numbers of women highlight shortcomings in current services. Overall, the results suggest a lack of equity in provision of adequate emotional and psychological support for women in pregnancy after stillbirth or neonatal death. For clinicians, ensuring adequate access to and reading of case notes before consultations is vital to avoid insensitive remarks and promote effective communication.

Psychological distress in pregnancy is associated with major adverse outcomes for mother and baby, including increased risk of perinatal mental illness which is acknowledged as a major public health concern. There is an urgent need for more research, particularly with socially disadvantaged groups, to improve understanding of parents’ needs and to enable the development of interventions to improve emotional support [34]. Improving continuity, particularly of midwifery care could potentially address many of the identified issues, but has yet to be evaluated in this context.

### Ethics

A favourable ethics opinion for the study was obtained through National Research Ethics Service (NRES) Committee North West – Greater Manchester North (Ref 11/NW/0806) and the University of Manchester Ethics Committee. Consent was confirmed by the participant completing and submitting or returning the questionnaire.

### Consent to publish

Not applicable.

### Availability of data and materials

Research governance approval conditions for the study prevent distribution of the full study dataset outside the named research team.

## Abbreviations

ADU: antenatal day unit
BME: black and minority ethnic
CTG: cardiotocograph
NMC: Nursing and Midwifery Council
NRES: National Research Ethics Service
RCOG: Royal College of Obstetricians and Gynaecologists
UK: United Kingdom
WHO: World Health Organisation
